# Oncological and Functional Outcomes after Hemicortical Resection and Biological Reconstruction Using Allograft for Parosteal Osteosarcoma of the Distal Femur

**DOI:** 10.1155/2022/5153924

**Published:** 2022-06-02

**Authors:** Olga D Savvidou, Stavros Goumenos, Ioannis Trikoupis, Angelos Kaspiris, Dimitra Melissaridou, Panagiotis Gavriil, Jimmy Georgoulis, Panayiotis J Papagelopoulos

**Affiliations:** ^1^First Department of Orthopedics, National and Kapodistrian University of Athens, Medical School, Zografou, Greece; ^2^ATTIKON University General Hospital, Rimini 1, Chaidari, Athens, Greece; ^3^Laboratory of Molecular Pharmacology, Group of Orthopaedic Research, Department of Pharmacy, School of Health Sciences, University of Patras, Patras, Rion 26504, Greece

## Abstract

**Background:**

Parosteal osteosarcoma (PAOS) is a surface osteosarcoma. Treatment options include wide excision and endoprosthetic or allograft. However, due to the low local recurrence and metastasis rate, when it appears in the posterior surface of the distal femur, the lesion can be managed with hemicortical wide resection and biological reconstruction with hemicortical allograft. The purpose of this study is to evaluate the oncological and functional outcomes of patients with parosteal osteosarcoma (PAOS) of the posterior cortex of the distal femur who underwent biological reconstruction after hemicortical resection.

**Methods:**

Eleven patients who underwent wide tumor resection and defect reconstruction of the posterior surface of the distal femur using hemicortical allograft were retrospectively studied. Local recurrence, metastasis, complications, and the functional outcome using the Musculoskeletal Tumor Society (MSTS) scoring system were evaluated.

**Results:**

The average postoperative follow-up period was 53.64 months (range, 30 to 84 months). At the latest follow-up, all patients had no evidence of disease without metastases. One patient with local recurrence underwent revision surgery with fibula autograft reconstruction. The mean MSTS score was 93.45 ± 3.56.

**Conclusions:**

Treatment of patients with PAOS of the posterior aspect of the distal femur with hemicortical resection and allograft reconstruction has satisfactory oncological and functional outcome and low complication rates.

## 1. Introduction

Osteosarcomas are high-grade intramedullary bone tumors. Limb salvage surgery can be achieved with wide excision and endoprosthetic reconstruction. Parosteal osteosarcomas (PAOSs) belong to surface osteosarcomas, a rare different clinicopathological entity of osteogenic tumors rather than a subtype of intramedullary osteosarcomas [1].

PAOSs are the most common surface tumor. They have predominance for young (2^nd^–4^th^ decade) females, accounting for 4% to 6% of the osteosarcomas. Although the posterior cortex of the distal femur is most commonly affected, it can also be detected in the metaphysis of other long bones [2].

Wide tumor resection and endoprosthesis or biological reconstruction are the most commonly used therapeutic approaches [2]. PAOSs are low-grade tumors with low risk for metastasis and local recurrence; hence, PAOS of the distal femur can be managed with hemicortical wide tumor excision and biological reconstruction, avoiding the risks of endoprosthetic reconstruction complications [3].

The purpose of this study was to evaluate the oncological and functional outcomes of hemicortical excision and biological reconstruction using allograft for the treatment of PAOS of the posterior aspect of the distal femur.

## 2. Materials and Methods

Clinical, surgical, radiological, and follow-up information of eleven patients with PAO of the posterior cortex of the distal femur that underwent wide hemicortical resection and reconstruction with hemicortical strut allograft in First Orthopaedic Department of “Attikon” University Hospital, Athens, Greece, from 2010 to 2018, were retrospectively collected. Only individuals that fulfilled the criteria defined by the World Health Organization (WHO) classification for Tumors of Soft Tissue and Bone [1] were included in the study.

Population characteristics such as age, sex, and BMI and tumor diagnostic features such as the primary location histological grading and presence of metastasis, type of surgical intervention and tumor surgical margins, and period of follow-up and local recurrence as well as functional and oncological outcomes were anonymously received from the medical archives of our department.

Finally, 7 females and 4 males were included in the study. The mean age and BMI of the patients were 29 years (Min: 16, Max: 41, and SD: 7.46) and 25 kg/m^2^ (Min: 19.6, Max: 28, and SD: 2.76), respectively. Tumor assessment was based on computer tomography (CT), magnetic resonance imaging (MRI), and histopathological results. Needle biopsy under CT-guided was undertaken in all patients. A coaxial technique was preferred as multiple cores could be obtained through a single bone window. CT scan provided information about the route and the precise location of neurovascular structures and assisted in safely avoiding the abovementioned vital structures that were not affected by the tumor. The choice of a needle biopsy method was based on the planned surgical approach, keeping the biopsy track excisable. Regarding the posterior biopsy, it was planned according to the site of the lesion, through a posteromedial or posterolateral approach.

All patients were diagnosed with well-differentiated PAO of the distal femur that involved less than 60% of the cortex circumference without intramedullary extension. None of the patients appeared distant metastatic disease (Table 1).

The research complies with the 1964 Helsinki Declaration and its later amendments, and it was approved by the Ethical Committee of ATTIKON University General Hospital with the reference number of AD 232/19-04-2021. All patients agreed to participate in the study and provided written consent prior to publication.

## 3. Operative Technique and Follow-Up

The operation was performed under a general anesthesia, and a pneumatic tourniquet was used on the proximal thigh. Depending on the location and the size of the lesion, 2 or 3 cortical compression screws or an anatomical distal femoral plate ± cortical screws were used. Needle track biopsy was excised in all cases.

When the tumor was small and totally posterior, the patient was in the prone position and a posterior approach was undertaken. Two or 3 cortical compression screws were used in the posterior-anterior direction to secure the allograft to the distal femur.

When the tumor was large in size with extension to the lateral or medial aspect of the distal femur, a lateral or medial approach was used and the patient was operated supine. A lateral or medial anatomical distal femoral plate ± cortical screws were used for prophylactic fixation.

**Figure 1:**
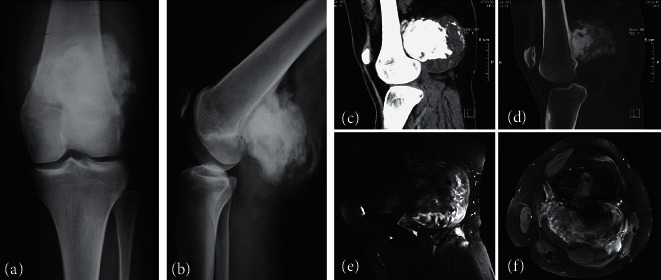
Parosteal osteosarcoma (PAO) in a 16-year-old female (no. 6). Anteroposterior (a) and lateral (b) radiographs of the knee showing a parosteal osteosarcoma as a large-ossified opacity attached to the posterior cortex of the distal femoral metaphysis. Sagittal computerized tomography (CT) reformatted images, soft tissue window (c), and bone window (d) demonstrate the characteristic separation between the tumor and the intact femoral cortex (cleft sign) as well as the ossified thick stuck lytic areas are seen within the ossified mass which is surrounded by a thick hypodense rim representing cartilaginous tissue. A fat-suppressed T2w magnetic resonance image sagittal (e) and axial view (f) showing the densely ossified stuck centrally, the inhomogenous moderately, T2 hyperintense mass in the middle, and the hyperintense cartilaginous component in the periphery. There is no intramedullary extension of the tumor.

**Figure 2:**
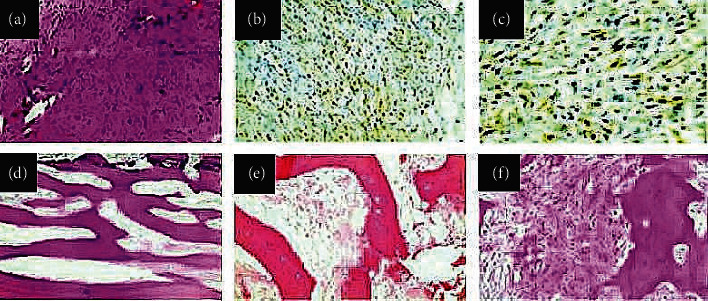
(a) Representative photomicrograph for histological evaluation with hematoxylin-eosin staining (original magnification: 200X): hypocellular tumor with spindle cells showing mild atypia arranged in fascicles in desmoplastic collagenous stroma. The histological results are compatible with parosteal osteosarcoma (PAOS). (b, c) Representative photomicrographs for immunohistochemical assessment displaying tumor cells positively immunostained for MDM-2 (original magnification: 400X) and CDK4 (original magnification: 400X), respectively, with nuclear location. (d) Representative photomicrograph of the slices for histological evaluation after tumor excision with hematoxylin-eosin staining (original magnification: 200X): parallel trabeculae of the well-formed woven bone with spindle neoplastic cells in collagenous stroma, while (e) the neoplastic population is characterized by mild cellularity and atypia (original magnification: 40X). (f) Representative photomicrograph for histological evaluation with hematoxylin-eosin staining showing clusters of moderate cellularity and moderate atypia in PAOS (original magnification: 40X).

**Figure 3:**
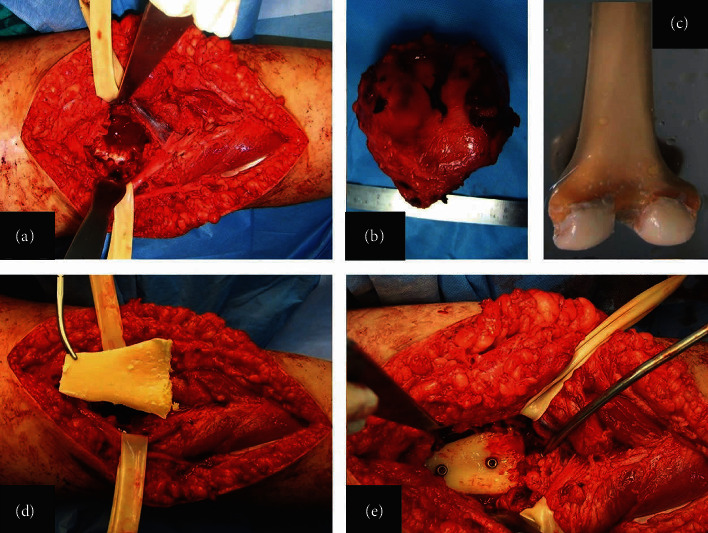
Intraoperative image of the popliteal fossa after tumor resection and identification of the tibia nerve and the popliteal artery (a). Hemicortical resection (b). The resected specimen (c). Distal femoral allograft (d). The part of the posterior femoral allograft matching the dimension of the posterior distal femoral defect after tumor excision (e). Two cortical screws were used to fix the allograft to the distal femur.

**Figure 4:**
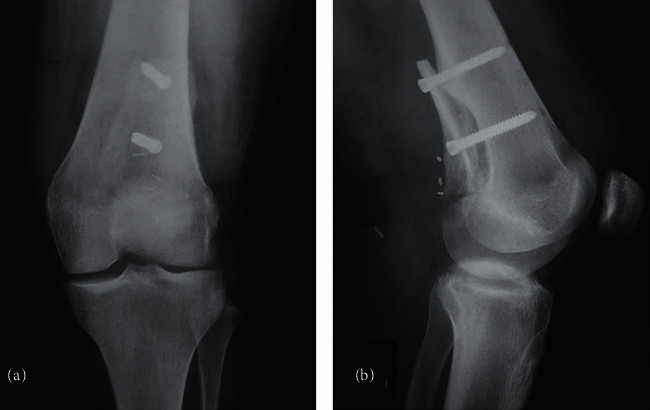
Anteroposterior (a) and lateral (b) radiographs 6 months postoperatively.

In 4 patients, a posterior approach was used, with the patient in the prone position. A longitudinal incision at the posterior aspect of the distal thigh and knee was performed. The fascia and the popliteal space were carefully dissected, and the peroneal and the tibial nerve as well as the femoral artery and vein were identified and preserved. The posterior aspect of the distal femur was exposed, and using a thin blade saw and osteotomes, the posterior cortex of the distal femur was resected. The posterior cortex of a distal femur allograft was prepared to match the cortex defect of the host. Due to the size of the tumor, 2 or 3 cortical compression screws were used in a posterior-anterior direction to secure the allograft to the host bone (Figures 1–4).

**Figure 5:**
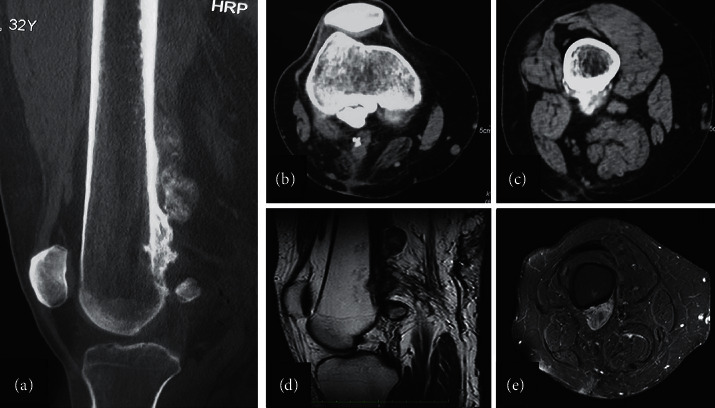
Computer tomography (CT) of the right femur in a 32-year-old male with parosteal osteosarcoma of the distal femur (a). Sagittal CT with multiple large-ossified masses adjacent to the posterior surface of the distal femur (b). Axial view displaying the tumor's broad-based stuck attached to cortical surface associated with dense central ossification and the cleft sign that separated the ground glass mass from the bone cortex, with no evidence of medullary involvement (c). Magnetic resonance imaging of the distal femur (sagittal (d) and axial (e) views) showing the round mass of low signal intensity on T1-weighted imaging. The cortex appears intact along the deep surface of the lesion.

**Figure 6:**
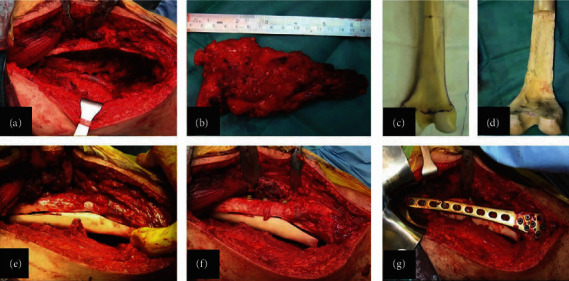
Intraoperative images showing (a) midline longitudinal incision at the posterior aspect of the distal femur and hemicortical resection. (b) Tumor resection. (c) Distal femur allograft. (d) Preparation of the allograft. (e) Matching the allograft to the bone deficit. (f) Allograft fixation with four cortical screws in the anteroposterior direction (g). The anatomical distal femoral plate is used as a bridging plate.

**Figure 7:**
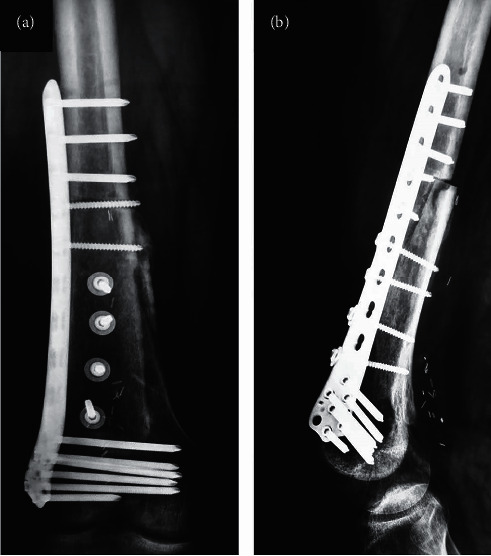
Anteroposterior (a) and lateral radiographs (b) nine months postoperatively showing incorporation of the allograft at the distal part. However, the proximal part of the allograft shows partial union.

In 7 patients, a lateral or medial approach was used, with the patient in the supine position. Meticulous dissection and identification of the neurovascular structures were undertaken, and the distal femur was exposed. Using a thin blade saw and osteotomes, the cortex of the distal femur with the tumor was resected. The cortex of a distal femur allograft was prepared to match the cortex defect of the host. An anatomic distal femoral plate (in 4 patients a lateral and in 3 patients a medial plate) ±  cortical screws were used for prophylactic fixation (Figures 5–7).

The size of resection was based on the preoperative imaging (CT and MRI) with at least 1 cm of tumor-free surgical margins. The mean cortical resection was 14.64 ± 5.5 cm.

A drain suction was used, and the wound was closed in layers. Postoperatively, a posterior splint and later a brace were applied until radiographical union was demonstrated on CT scan. Partial weight-bearing was started when more than 50% union of the transverse and longitudinal osteotomies appeared in the radiographic evaluation 6–12 weeks after operation, while full-weight bearing was allowed when allograft incorporation was achieved (average time 7.64 months (Min:6, Max: 12, SD: 1.81).

None of the patients were lost during the follow-up. The average postoperative follow-up was 53.64 months (Min: 30, Max: 84, and SD: 16.94). None of the patients received neoadjuvant chemotherapy.

All surgical procedures, postoperative evaluation, and assessment were performed by the same surgical team. The primary endpoint of our study was the achievement of satisfactory disease control. Secondary endpoints were as follows: functional outcomes during the last follow-up, time to graft incorporation, and assessment of postoperative complications.

As for the primary endpoint assessment, all patients underwent sequential imaging staging (MRI) and computer tomography (CT) scan for local and systematic disease progression and a CT scan of the chest every four months in the first two years and every six months thereafter. Regarding the secondary endpoints of our study, functional results were evaluated using the Musculoskeletal Tumor Society (MSTS) score [4] and measuring the knee range of motion.

The time of the allograft incorporation was established based on radiographic and CT scan findings of union. The patients were examined every month until the osteotomies had consolidated and then after every six months. Bridging across three of four cortices in biplanar radiographs and CT scan findings were considered evidence of consolidation.

Pathology specimens were evaluated to assess the adequacy of surgical margins. A wide surgical margin procedure was documented when a cuff of normal tissue totally covered the tumor. A marginal margin was recognized when a free margin of the normal cortex and the marrow was seen microscopically between the tumor and the bone^20^.

The postoperative complications were classified as mechanical (types 1, 2, and 3) and nonmechanical (types 4 and 5) according to the classification system regarding the failure of limb salvage after biological reconstruction described by Henderson et al. [5].

## 4. Results

Wide excision of the lesion with clear surgical margins was achieved in all patients based on histopathological examination. At the follow-up examination, no distant metastases were detected. All patients had no evidence of disease at the latest follow-up evaluation (primary endpoint).

The mean MSTS score was 93.45 (Min: 88, Max: 99, and SD: 3.56). All patients were ambulatory without any postoperative pain or restriction of daily activities at the latest follow-up. The mean range of knee flexion was 105.1° (Min: 95, Max: 118, SD: 7.66).

Radiologic findings showed successful union within the first postoperative year. The average time of allograft incorporation was 7.64 months (Min:6, Max: 12, and SD: 1.81).

**Figure 8:**
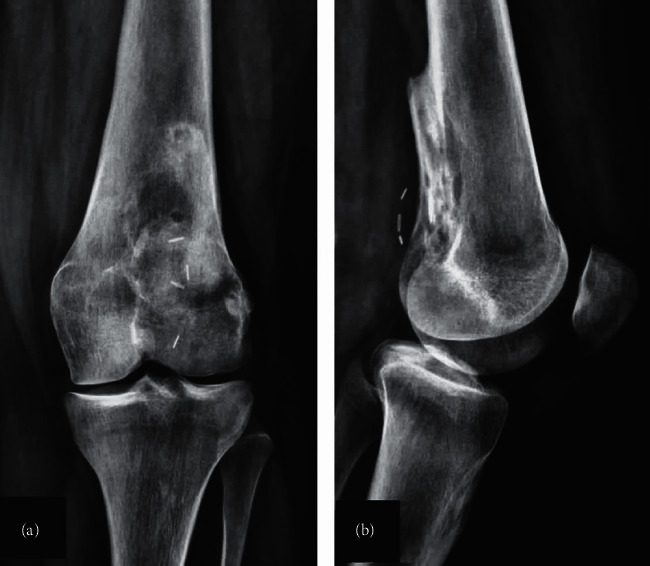
Parosteal osteosarcoma (PAO) in a 16-year-old female (no. 6). Anteroposterior (a) and lateral (b) radiographs four years after operation showing recurrence of PAO.

**Figure 9:**
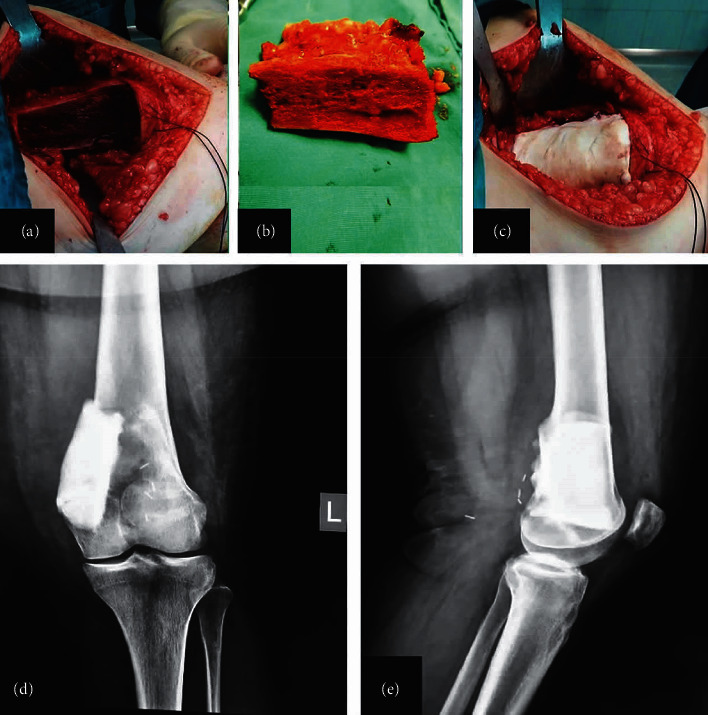
Intraoperative images with complete excision of the posterior-medial part of the distal femur (a), the excised specimen (b), and polymethyl-methacrylate (PMMA) used to fill the osseous void (c). Postoperative radiographs of anteroposterior (d) and lateral (e) views showing the PMMA in the posterior-medial part of the distal femur.

**Figure 10:**
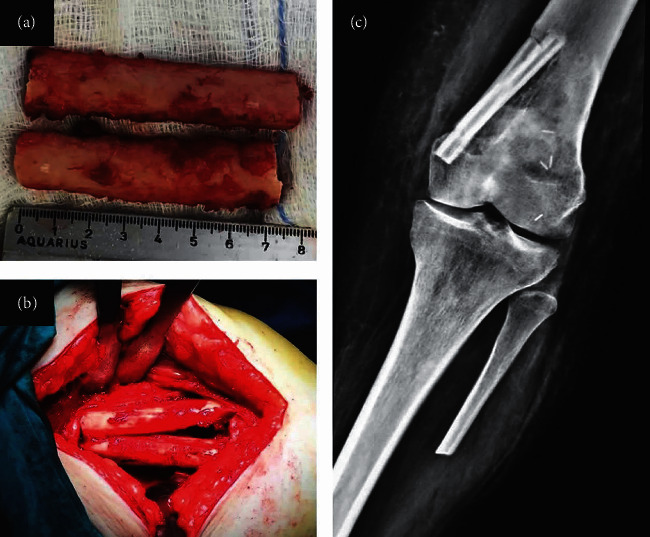
(a) Autograft from the contralateral fibula was used and was cut in the longitudinal axis. (b) Two pieces of hemicortical fibula were inserted to the posterior-medial part of the distal femur after excision of the PMMA. (c) Postoperative anteroposterior radiographs of the knee showing the fibula autografts in place.

**Figure 11:**
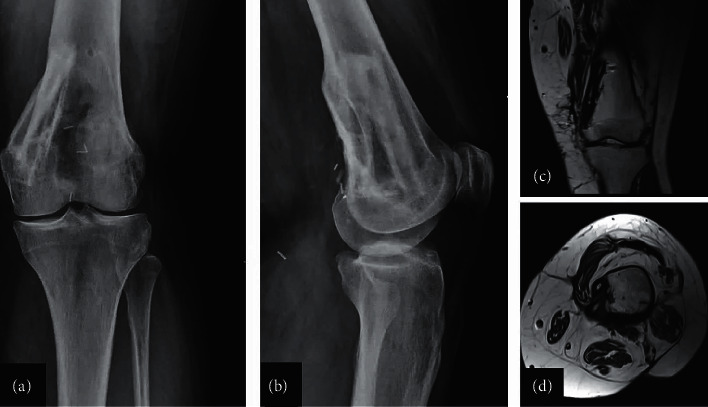
Anteroposterior (a) and lateral (b) radiographs of the knee 13 months postoperatively showing incorporation of the fibula autografts with no signs of recurrence. Magnetic resonance imaging of the distal femur (sagittal (c) and axial (d) views) showing the distal femur with the fibula autografts with no signs of recurrence, infection, or autograft fracture.

As far as the postoperative complications, there were no soft tissue (type 1), hardware failure (type 3), or implant-related infections (type 4) based on the Henderson criteria. Only one patient had local recurrence (case no. 6) and underwent revision surgery with wide excision and reconstruction using fibula autografts (Figures 8–11). This patient remains free of the disease five years after operation (Table 1**)**.

## 5. Discussion

Despite the fact that PAO is the most frequent surface osteosarcoma, it is a rare bone tumor [6]. It is considered a low-grade tumor that originates from the periosteum and appears with low risk for local recurrence and metastases. The demographic characteristics of the patients included in our study are in agreement with the findings of the international literature as PAOS was primarily developed in young adult female patients. Indeed, the incidence of PAOS is prominent in female individuals in their third decade of life [6]. Moreover, studies reported that the most common location of the tumor is the posterior surface of the distal femoral metaphysis followed by the proximal tibial and femoral metaphysis being in line with our results. Symptoms include a slow-growing painless mass at the beginning. During time, as the mass increases, the pain worsens. If the tumor is closed to a joint, the decreased range of motion might occur [7, 8].

Radiographically, the lesion appears as a large-lobulated mass with large usually central bone formation and a thick mineralized stalk fixed onto the bone surface, without extension into the medullary cavity.

Despite the fact that needle biopsy shows lower accuracy than cytogenetic, molecular, immunohistochemical, or FISH (fluorescence in situ hybridization) studies, in all of our 11 cases, the combination of clinical, radiological, and needle biopsy findings provided efficient diagnostic results. Histological and immunohistochemical analysis revealed that the tumor was composed of hypocellular areas of spindle cells arranged in fascicles in desmoplastic collagenous stroma with parallel trabeculae of the well-formed woven bone. Spindle cells were characterized by minimal, or less frequently, moderate, atypia and limited mitotic activity.

PAO demonstrates 90% survival at 10-year follow-up after wide resection [5]. After wide tumor excision, endoprosthesis or different reconstruction techniques can be used. In our study, all patients were diagnosed with early-stage PAOS and were treated with hemicortical resection and biological reconstruction with the application of allograft. Moreover, prophylactic plating fixation was used in seven patients. The main indications of plate utilization were the relatively localized tumor, the cortical involvement, and the limited soft tissue expansion [9–13]. We must highlight the fact that in patients with PAOS of the distal femur, due to the low grade of the tumor and the young age of the patients, hemicortical resection and reconstruction of the bone defect using polymethyl-methacrylate (PMMA) or biological materials, such as fibular autografts, allografts, or pasteurized/autoclaved/irradiated host bones, with or without prophylactic fixation were correlated with good oncological and functional outcome [10–13].

On the contrary, endoprosthetic replacements of the femoral defect after PAOS resection have demonstrated long-term survival and good functional outcome [14], but they were linked to the increased postoperative complication rate including infection, aseptic loosening, mechanical failure, fracture of the prosthesis, or the adjacent bone [2, 15, 16]. Similarly, application of allografts allows mechanical and biological reconstruction after tumor excision, but they are, also, associated with high rates of complications, including fracture, nonunion, transmission of disease, and infection [17].

Hemicortical resection of the distal femur in the early stage of PAOS of the distal femur was first described by Campanacci et al. in 1984 [18]. Later, this method was established as a safe therapeutic procedure [10]. However, due to the rarity of patients with PAO, there are limited studies with efficient data that examined the functional outcomes of this surgical intervention. Our results analysis demonstrated satisfactory oncological and functional results. Specifically, the mean MSTS and the range of knee flexion scores were 93.45 and 105.1°, respectively. Our findings are in agreement with many studies that reported satisfactory functional and oncological outcomes after hemicortical resection of PAOS of the distal femur. In particular, Lewis et al. reported very good oncological and functional outcomes without any complications using this technique in 6 patients with PAOS of the distal femur [11]. Deijkers et al. analyzed 22 allograft hemicortical reconstruction procedures, also showing very satisfying oncological and functional outcomes. All patients had good or excellent MSTS scores that were accompanied by low complication rates. Furthermore, the incorporation rate of allograft in thirty months was observed in 100% postoperatively [12]. Agarwal et al. reviewed 10 patients who underwent hemicortical excision of the distal femur and reconstruction with autograft or allograft. In both methods, the functional outcome was optimal without any major surgical complications or local recurrence [3]. Liu et al. also reported very good outcomes with relative low complication rates in 13 patients diagnosed with PAOS and managed with wide excision and reconstruction with pasteurized hemicortical autograft and internal fixation. The authors stated that the technique, although was operational demanding, was safe and effective in selected patients [19].

Compared to endoprosthetic reconstruction after wide resection of PAOS of the distal femur, many studies demonstrate, respectively, good functional and oncological outcomes. Funovic et al. compared 12 patients who underwent prosthetic reconstruction, with 11 patients who underwent biological reconstruction. The authors concluded that the oncological and functional outcome was not altered between the two reconstruction methods [20]. Nevertheless, the increased rate of revision arthroplasty (58%) in the endoprosthetic reconstruction group was observed when it was compared to the biologic reconstruction group (18%) at 10 years postoperatively [20]. In addition, Wilke et al. reported that the comparison between 5 and 7 patients who underwent endoprosthetic and biological reconstruction with allograft application, respectively, revealed the same mean MSTS score (mean MSTS 23) and the similar rate of complications and reoperation between the two groups [2].

Although in our study the application of hemicortical allograft was not correlated with any complications supporting the evidence that it is a reliable biological reconstruction method after wide excision of PAOS of the posterior cortex of the distal femur, complications may occur. The most common complication is host bone fracture and local recurrence, followed by nonunion and infection. Rarely allograft fractures can occur. These complications are associated directly with the size of the bone defect after wide excision and often require surgical reintervention [21]. None of our patients had a host bone fracture, allograft fracture, nonunion, or infection. Local recurrence was observed in one patient and was managed with revision, and biological reconstruction with fibular autograft. This complication corresponded to the 9.1% of the patients included in the study being significantly lower than the rate of 17% that was referred in previous reports of the literature [22]. In addition, the revision intervention of this patient was associated with very good functional results avoiding the application of endoprosthetic replacement. Although the frequency of local recurrence was not correlated with the surgical intervention or the histological grade of the tumor [22], inadequate wide resection and dedifferentiated PAOS have been described as potential negative predictors for its relapse [9, 22–25].

While hemicortical resection and allograft of the PAO of the posterior aspect of the distal femur avoid complications associated with wide distal femur resection and reconstruction such as endoprosthesis, drawbacks such as a higher risk of a positive margin or recurrence should be addressed. Currently, three-dimensional measurements on the tumor before surgical resection using computer technology and establishing a 3-dimensional model and a 3D printing osteotomy guide plate could assist in accurately resecting the tumor lesion [23].

The results of this case series are in accordance with those of the published literature. We acknowledge that despite the satisfying results, the study has several limitations. It is an observational, retrospective study, which included a small number of patients, without a comparison group. However, all 11 patients were operated by the same surgeon (PJP). Randomized multicenter studies with a large number of patients with PAOS of the distal femur are needed in order to establish these functional and oncological results and compare them to the other reconstruction techniques.

## 6. Conclusion

Parosteal osteosarcoma of the posterior surface of the distal femur without intramedullary involvement can be treated with hemicortical excision and biological reconstruction with hemicortical allograft. This procedure is safe and reliable with good oncological and functional results accompanied by low complication rates.

## Figures and Tables

**Table 1:** Clinical and demographic characteristics of the included patients.

Patient	Gender	Age (years)	BMI (kg/m^2^)	Follow-up (months)	Resection Length (cm)	Osteosynthesis	MSTS score	Range of flexion (degrees)	Complication type (Henderson)	Time to union (months)<
1	M	24	22.4	30	23	Plate + screws	95	95	—	7
2	M	19	26.7	32	14	Plate + screws	97	111	—	8
3	F	32	21	40	19	Plate + screws	94	98	—	6
4	F	27	27	44	9	Screws	91	102	—	6
5	F	31	24	49	20	Plate + screws	89	100	—	9
6	F	16	20.8	84	11	Screws	90	97	Type 5 (recurrence)	12
7	F	20	28	58	8	Screws	96	107	—	6
8	F	38	19.6	61	17	Plate + screws	93	108	—	7
9	M	41	23	68	20	Plate + screws	88	104	—	7
10	F	35	25.2	72	13	Plate + screws	96	116	—	9
11	M	34	27	52	7	Screws	99	118	—	7

## Data Availability

The data used to support the findings of this study cannot be shared.

## References

[1] WHO (2020). WHO Classification of tumors. *Soft tissue and bone tumours*.

[2] Wilke B. K., Cooper A. R., Gibbs C. P., Scarborough M. T., Spiguel A. R. (2018). Long-term functional outcomes of distal femoral replacements compared to geographic resections for parosteal osteosarcomas of the distal femur. *The Iowa Orthopaedic Journal*.

[3] Agarwal M., Puri A., Anchan C., Shah M., Jambhekar N. (2007). Hemicortical excision for low-grade selected surface sarcomas of bone. *Clinical Orthopaedics and Related Research*.

[4] Bolia I. K., Savvidou O. D., Kang H. P. (2021). Cross-cultural adaptation and validation of the musculoskeletal tumor society (MSTS) scoring system and toronto extremity salvage score (TESS) for musculoskeletal sarcoma patients in Greece. *European Journal of Orthopaedic Surgery and Traumatology*.

[5] Henderson E. R., O’Connor M. I., Ruggieri P. (2014). Classification of failure of limb salvage after reconstructive surgery for bone tumours: a modified system Including biological and expandable reconstructions. *The Bone & Joint Journal*.

[6] Hang J. F., Chen P. C. H. (2014). Parosteal osteosarcoma. *Archives of Pathology and Laboratory Medicine*.

[7] Nouri H., Ben Maitigue M., Abid L. (2015). Surface osteosarcoma: clinical features and therapeutic implications. *Journal of Bone Oncology*.

[8] Gholamrezanezhad A., Basques K., Kosmas C. (2018). Peering beneath the surface: juxtacortical tumors of bone (part II). *Clinical Imaging*.

[9] Zaikova O., Grimer R. J., Kindblom L. G. (2018). Parosteal osteosarcoma: risk factors for local recurrence and death. *Journal of Bone Joint Surgery British volume*.

[10] Enneking W. F., Springfield D., Gross M. (1985). The surgical treatment of parosteal osteosarcoma in long bones. *Journal of Bone Joint Surgery*.

[11] Lewis V. O., Gebhardt M. C., Springfield D. S. (2000). Parosteal osteosarcoma of the posterior aspect of the distal part of the femur: oncological and functional results following a new resection technique. *Journal of Bone and Joint Surgery American Volume*.

[12] Deijkers R. L. M., Bloem R. M., Hogendoorn P. C. W., Verlaan J. J., Kroon H. M., Taminiau A. H. M. (2002). Hemicortical allograft reconstruction after resection of low-grade malignant bone tumours. *Journal of Bone and Joint Surgery British Volume*.

[13] Prabowo Y., Kamal A. F., Kodrat E., Prasetyo M., Maruanaya S., Efar T. S. (2020). Parosteal osteosarcoma: a benign-looking tumour, amenable to a variety of surgical reconstruction. *International journal of surgical oncology*.

[14] Kavanagh T. G., Cannon S. R., Stoker D., Stoker D. J., Kemp H. (1990). Parosteal osteosarcoma. Treatment by wide resection and prosthetic replacement. *Journal of Bone Joint and Surgery British volume*.

[15] Guo W., Ji T., Yang R., Tang X., Yang Y. (2008). Endoprosthetic replacement for primary tumours around the knee: experience from Peking University. *Journal of Bone Joint and Surgery British volume*.

[16] Gosheger G., Gebert C., Ahrens H., Streitbuerger A., Winkelmann W., Hardes J. (2006). Endoprosthetic reconstruction in 250 patients with sarcoma. *Clinical Orthopaedics and Related Research*.

[17] Ozaki T., Hillmann A., Bettin D., Wuisman P., Winkelmann W. (1997). Intramedullary, antibiotic loaded cemented, massive allografts for skeletal reconstruction. 26 cases compared with 19 unce¬mented allografts. *Acta Orthopaedica Scandinavica*.

[18] Campanacci M., Picci P., Gherlinzoni F., Guerra A., Bertoni F., Neff J. R. (1984). Parosteal osteosarcoma. *Journal of Bone Joint and Surgery British volume*.

[19] Liu T., Liu Z.-Y., Zhang Q., Zhang X.-S. (2013). Hemicortical resection and reconstruction using pasteurised autograft for parosteal osteosarcoma of the distal femur. *The Bone & Joint Journal*.

[20] Funovics P. T., Bucher F., Toma C. D., Kotz R. I., Dominkus M. (2011). Treatment and outcome of parosteal osteosarcoma: biological versus endoprosthetic reconstruction. *Journal of Surgical Oncology*.

[21] Bus M. A., Bramer J. M., Schaap G. R. (2015). Hemicortical resection and inlay allograft reconstruction for primary bone tumors. *Journal of Bone Joint and Surgery*.

[22] Laitinen M., Parry M., Albergo J. I. (2015). The prognostic and therapeutic factors which influence the oncological outcome of parosteal osteosarcoma. *Bone & Joint Journal*.

[23] Wu H., Yang S., Liu J. (2021). 3D printing quide plate for accurate hemicortical bone tumor resection in metaphysis of distal femoral: a technical note. *Journal of Orthopaedic Surgery and Research*.

[24] Savvidou O., Papakonstantinou O., Lakiotaki E. (2021). Surface bone sarcomas: an update on current clinicopathological diagnosis and treatment. *EFORT Open Reviews*.

[25] Walczak B. E., Johnson C. N., Howe B. M. (2015). Myositis ossificans. *Journal of the American Academy of Orthopaedic Surgeons*.

[26] https://www.researchsquare.com/article/rs-1119930/v1.

